# Cervical sagittal parameters were closely related to Neck Disability Index score after anterior cervical decompression and fusion

**DOI:** 10.1186/s13018-020-01836-x

**Published:** 2020-08-14

**Authors:** Yefu Xu, Sangni Liu, Feng Wang, Xiaotao Wu

**Affiliations:** 1 Department of Spine Surgery, Zhongda Hospital, No. 87 Dingjiaqiao, Gulou District, Nanjing, 210009 China; 2 School of Medicine, Southeast University, Nanjing, 210009 China

**Keywords:** Anterior cervical decompression and fusion (ACDF), Cervical spondylotic myelopathy (CSM), Cervical sagittal parameters

## Abstract

**Background:**

ACDF treatment of CSM is currently recognized as a surgical method with reliable efficacy. However, the cervical radiographic findings in a certain group of patients showed that the symptoms were not completely relieved. This study will investigate the relationship between cervical parameters and prognoses after ACDF surgery.

**Methods:**

This study collected cases of CSM treated with ACDF in Zhongda Hospital from May 2014 to June 2018. The investigators recorded gender, age, cervical sagittal parameters, fusion segment, BMI, symptom duration, and NDI score. To compare the changes of parameters after surgery and explore the correlation between each factor and NDI score.

**Results:**

Generally, cervical lordosis increased and TS-CL decreased after surgery and during follow-up. Postoperative T1S, SVA and SCA decreased significantly compared to preoperative. T1S was positively correlated with CL (*r* = 0.245), SVA (*r* = 0.184), and negatively correlated with SCA (*r* = − 0.314) and NT (*r* = − 0.222). The last follow-up NDI score was positively correlated with T1S (*r* = 0.689), SVA (*r* = 0.155), TS-CL (*r* = 0.496), and age (*r* = 0.194), while negatively correlated with SCA (*r* = − 0.142). A linear regression model was established with the following formula: NDI = 0.809 × (T1S) − 0.152 × (CL) + 1.962 × (Sex) + 0.110 × (Age). T1S (*B* = 0.205, *P* < 0.001), CL (*B* = − 0.094, *P* = 0.041), and NT (*B* = 0.142, *P* = 0.023) were independent risk factors that affected whether the last follow-up NDI score was greater than preoperative.

**Conclusions:**

In ACDF treatment of CSM, there exists a close correlation between cervical sagittal parameters and NDI scores. T1S, CL, sex, and age were linearly dependent on NDI scores. The increase of T1S, NT, and the decrease of CL were risk factors that affected follow-up NDI score greater than preoperative. Reducing T1S is beneficial to clinical recovery.

## Introduction

The sagittal balance of the physiologically upright spine enables the intervertebral alignment to be maintained with minimal energy expenditure. Overloading of the cervical endplate can accelerate the degeneration of the spine, leading to cervical deformities that can lead to spinal cord compression and spinal cord tension [1]. Cervical spondylotic myelopathy (CSM) is one of the most common and harmful diseases in spine degenerative diseases, with the characteristics of concealment and intermittency. Anterior cervical decompression and fusion (ACDF) is an effective method for the treatment of cervical degenerative diseases.

ACDF can relieve symptoms by decompressing and releasing the spinal cord or nerve roots, which can effectively improve uncomfortable symptom. However, some patients still have postoperative symptoms of neck discomfort, sensation, and muscle strength decline. Researchers [2, 3] found that the patients with poor surgery effect, postoperative X-ray show up cervical lordosis (CL) loss, cervical sagittal vertical axial (cSVA), and thoracic 1 slope (T1S) oversize. They believed that cervical sagittal parameters were correlated with surgical outcomes and can affect prognostic.

NDI score is a common method to evaluate the surgical efficacy. Compared with other methods, NDI score is more comprehensive. It can reflect the recovery of neck function, the quality of patients’ daily life, and the remission of pain. Although previous studies have found correlations between NDI score and CL, SVA and T1S, there were few studies that had explored the relationship between follow-up NDI score deterioration and cervical parameters. In this study, we analyzed linear regression between NDI and cervical parameters, and explore the risk factors of increased NDI score after surgery. We expect to find the cervical parameters that were valuable for evaluation of surgical prognosis.

## Materials and methods

### Subjects

A retrospective study was conducted on cervical spondylotic myelopathy (CSM) patients treated with ACDF in spine surgery department of Zhongda Hospital from May 2014 to June 2018. Inclusion criteria and exclusion criteria are shown in Table 1. A total of 212 patients met the above criteria and were included in this study. Data were collected including gender, age, cervical sagittal parameters, fusion segment, body mass index (BMI), symptom duration, and NDI score.

**Table 1:** Inclusion criteria and exclusion criteria

Inclusion criteria	exclusion criteria
Typical signs and symptoms of spinal cord compression, combined with corresponding MRI findings.	previous history of cervical surgery, clear history of trauma, spinal tumor, infection, ankylosing spondylitis, congenital deformity.
The patients treated with ACDF in our hospital, with complete data and clear anatomical markers in X-ray image.	incomplete clinical and imaging data
Follow-up time more than 1 year	no imaging data of postoperative follow-up.

### Measures

#### Radiographic measurements

**Fig. 1:**
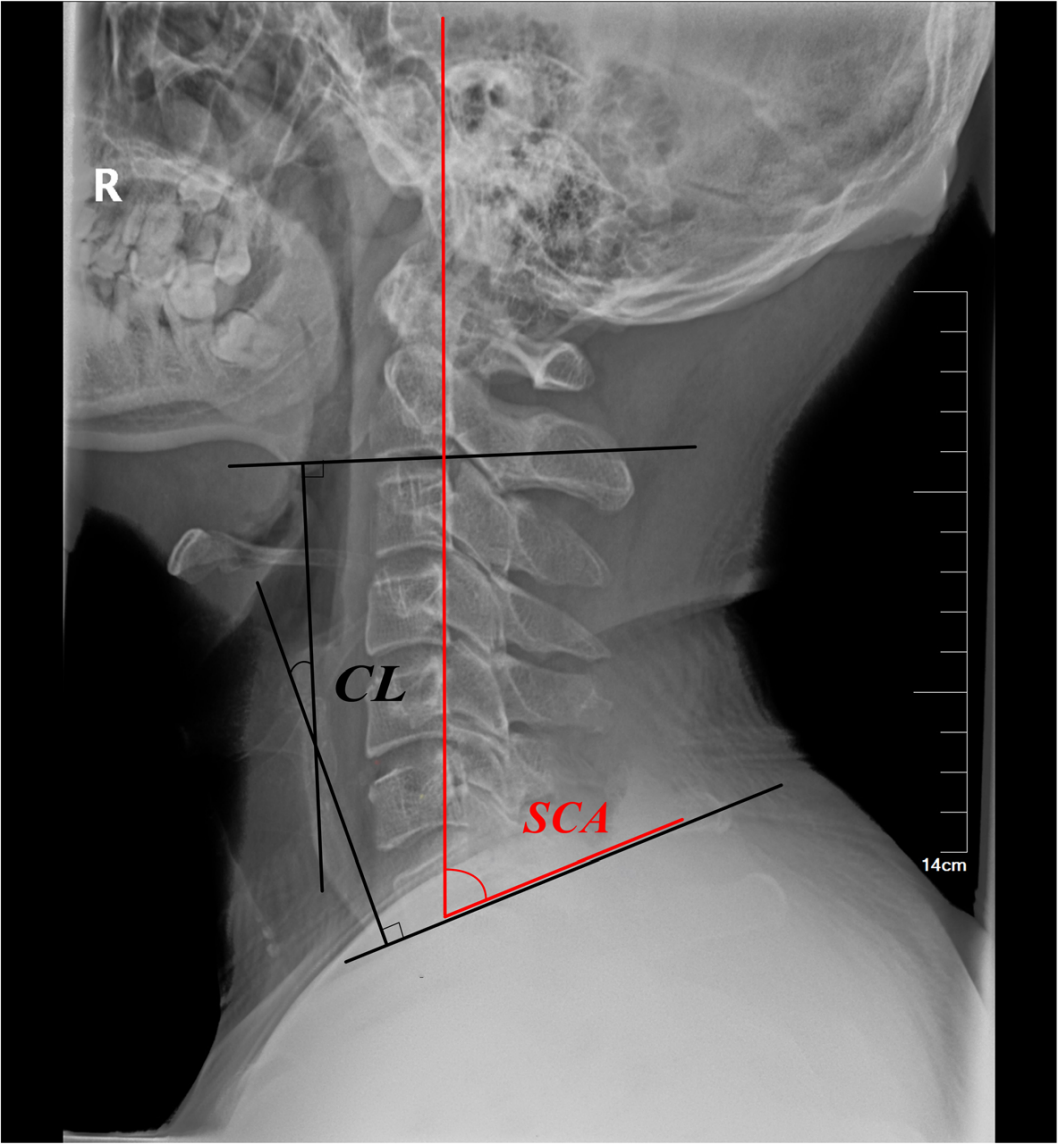
Method for measuring C2-C7 lordosis, Spine-cranial angle

**Fig. 2:**
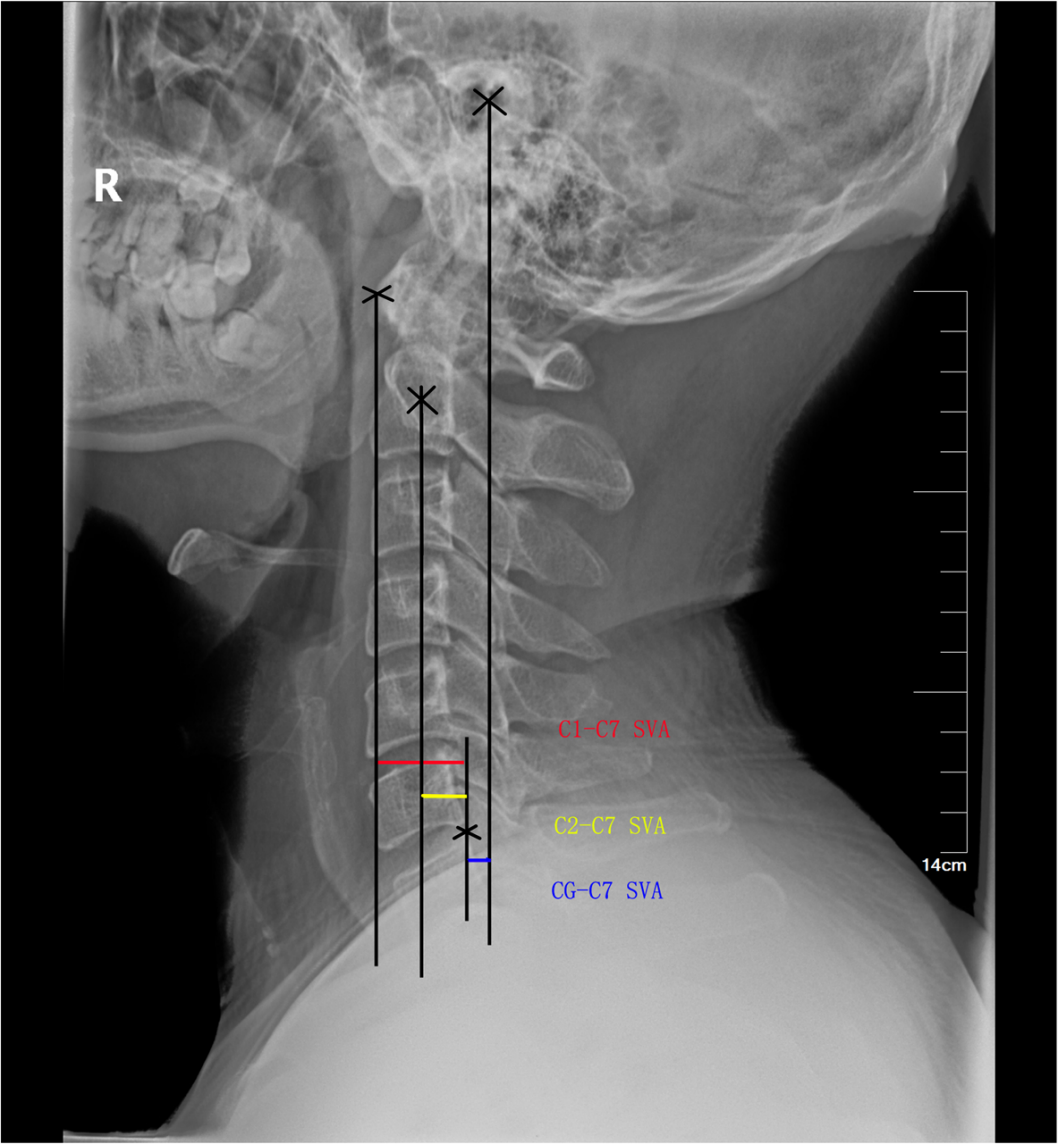
Method for measuring C2-7 SVA, C1-7 SVA and CG-C7 SVA

**Fig. 3:**
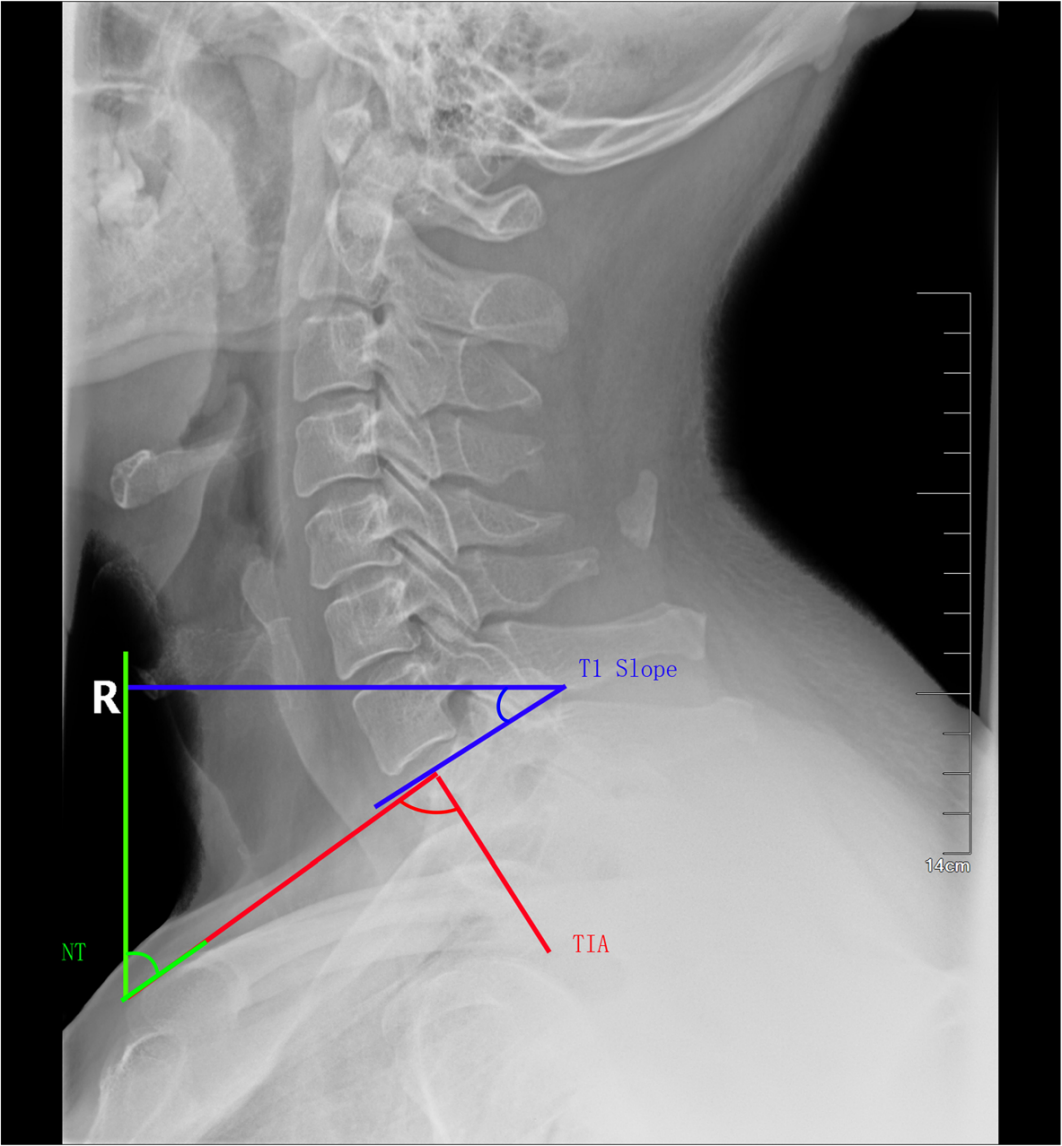
Method for measuring T1S, NT and TIA

Lateral cervical X-ray examination: the patients maintain horizontal gaze with their neck in the center. Measurement parameters include C2–7 Cobb angle, C2–7 sagittal vertical axial (SVA), thoracic 1 slope (T1S), thoracic 1 slope minus cervical lordosis (TS-CL), thoracic inlet angle (TIA), neck tilt (NT), and spine-cranial angle (SCA). The measurement method of cervical parameters is shown in Figs. 1, 2 and 3 and Table 2. All parameters were measured twice independently by two surgeons. The two measurements are averaged and recorded. Imaging data collection was divided into three stages: preoperative (on admission), postoperative, and follow-up (last outpatient visit).

**Table 2:** measurement methods of each parameter

	Definition of all parameters used: parameters description
C2–C7 lordosis	Angle between the lower plate of C2 and the lower plate of C7
C2–C7 SVA	The distance from the posterior, superior corner of C7 to the plumb-line from the centroid of C2
T1 slope	Angle between a horizontal line and the superior endplate of T1
TS–CL	Mismatch between T1 slope and CL
Thoracic inlet angle	Angle formed by a line perpendicular to the superior endplate of T1 and a line connecting the centre of the T1 upper endplate and the upper end of the sternum
Neck tilt	Angle formed by the reference vertical line drawn in the upper end of the sternum and a line connecting the centre of the T1 upper end plate and the upper end of the sternum
Spine-cranial angle	The angle is defined between the C7 slope and the straight line joining the middle of the C7 end plate and the middle of the sella turcica

#### Patient characteristics measures

Gender, age, fusion segments, BMI, and symptom duration were collected through the hospital record system. NDI scores were collected at preoperative and the last follow-up. The patient voluntarily filled in the NDI scores scale, and the total score was calculated by researchers. The NDI score was used to evaluate neck function and surgical outcome

### Statistical analysis

SPSS 21.0 (SPSS, Chicago) statistical software was used for statistical analysis of the measurement results. Descriptive analysis of preoperative, postoperative and follow-up parameters was conducted. One-way ANOVA was used for comparison. Independent sample *t* test was used to compare preoperative and follow-up NDI scores. Pearson analysis was used for inter-parameter correlation analysis and correlation between NDI scores and each factor. The linear regression equations of various factors and follow-up NDI scores were analyzed. *P* value less than 0.05 has a statistically significant difference. Logistic analysis was conducted for all data and follow-up NDI score. Whether the last follow-up NDI score was greater than preoperative was taken as the assessment result. All data were analyzed by binary logistic.

## Result

### Clinical outcome

**Fig. 4:**
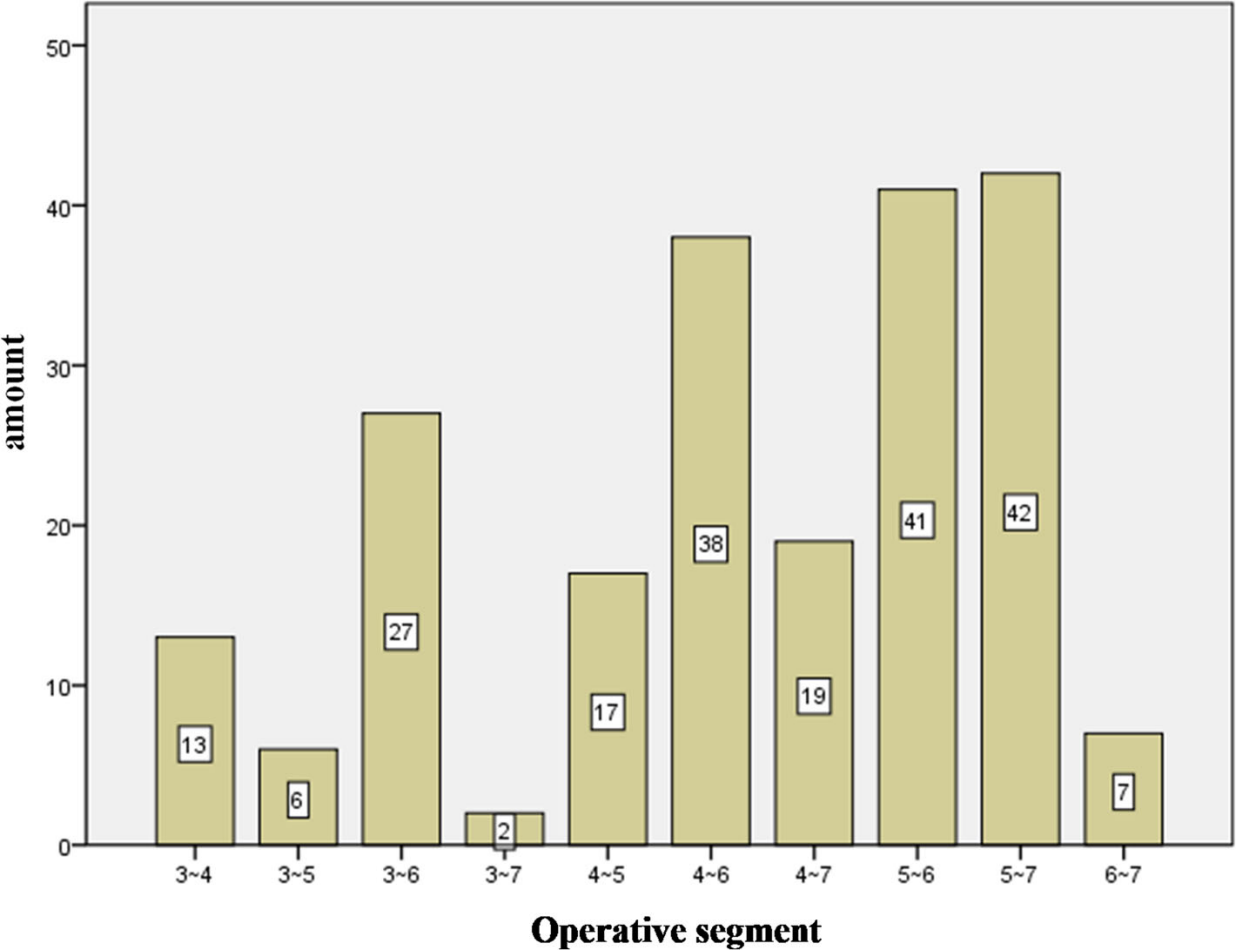
Amount of cases in operative segment

In this study, a total of 212 patients were included that mean NDI score follow-up time were 14.72 ± 5.93 months. There were 147 male patients and 65 female patients, with an average age of 52.59 ± 10.46 years and an average BMI of 23.78 ± 2.62 kg/m^2^. Seventy-eight cases underwent single-segment ACDF and 134 cases underwent multi-segment ACDF. Amount of cases in operative segment was shown in Fig. 4. Duration of clinical symptoms was 9.38 ± 16.95 months. The preoperative NDI score was 20.84 ± 6.17, and the last follow-up NDI score was 16.52 ± 6.90, which was significantly lower than preoperative (*P* < 0.001), the difference was statistically significant.

### Changes of cervical sagittal parameters after surgery

**Fig. 5:**
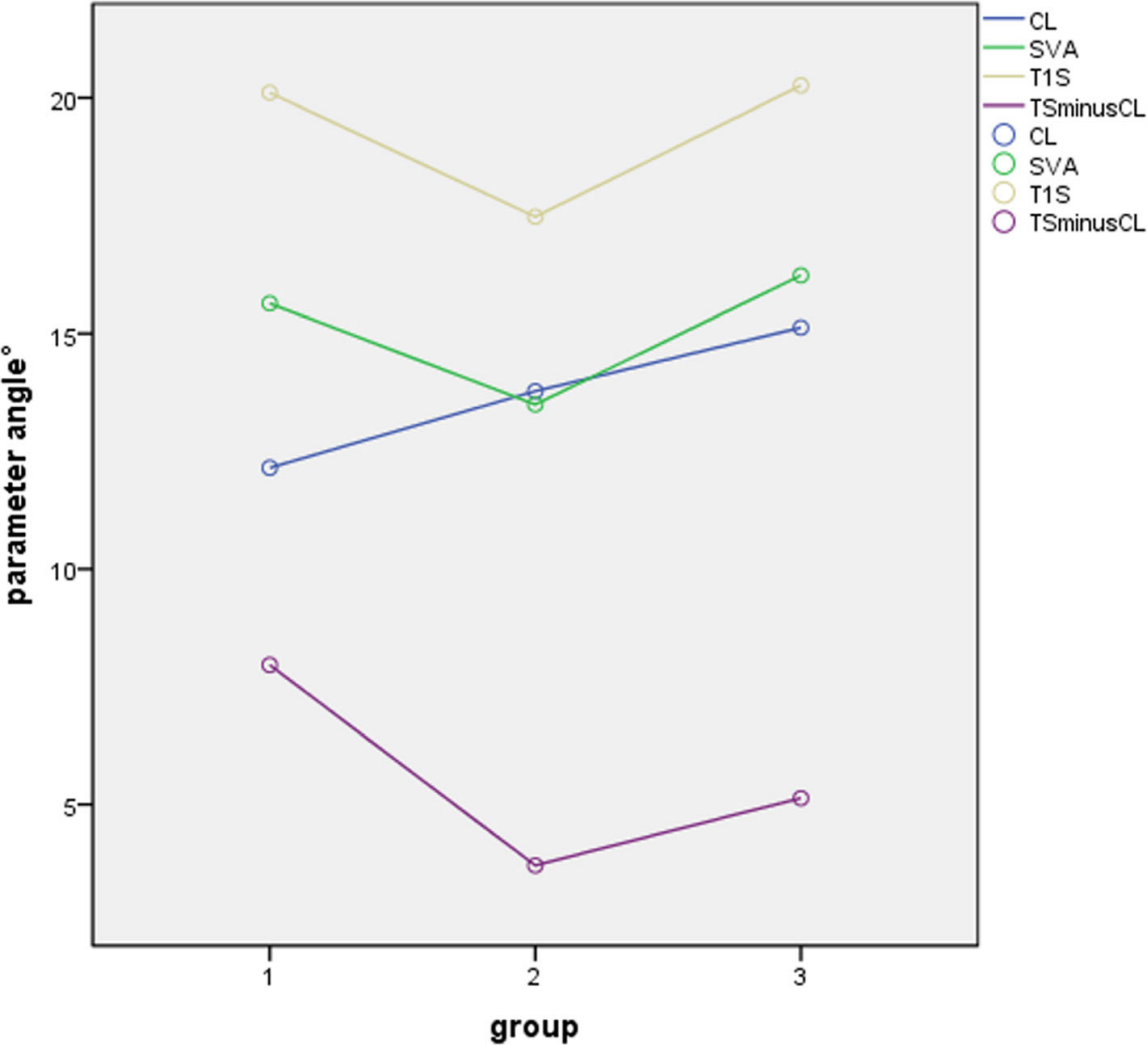
The changes of radiographic parameters after ACDF

The cases were divided into single-segment group and multi-segment group for comparison of parameters and NDI scores. The result is shown in Table 3. In the comparison of all cases, cervical lordosis was increased immediately after surgery (*P* = 0.040) and at the last follow-up (*P* < 0.001) compared with preoperative. TS-CL was decreased after surgery (*P* < 0.001) and at the last follow-up (*P* = 0.001) compared with preoperative (Fig. 5). Postoperative T1S (*P* < 0.001), SVA (*P* = 0.042), and SCA (*P* = 0.004) decreased significantly compared with that before surgery, the difference was statistically significant. However, there were no significant differences in SCA (*P* = 0.965), T1S (*P* = 0.991), and SVA (*P* = 0.978) during follow-up compared with those before surgery. NT and TIA had no change after surgery and during follow-up.

**Table 3:** Comparison of cervical parameters between single-segment group and multi-segment group

		Total (n=212)	Single-segment (n=78)	Multi-segment (n=134)	P
	Preoperative	11.17±8.56	11.69±8.51	12.42±8.55	.552
	Postoperative	13.30±7.53	14.15±8.04	13.56±7.21	.583
CL	Follow-up	14.64±6.44	15.33±6.42	15.01±6.46	.738
	P	0.040	.183	.557	
	P’	<0.001	.009	.016	
	Preoperative	15.64±9.12	15.22±10.25	15.88±8.42	.616
	Postoperative	13.49±7.09	13.80±8.47	13.31±6.18	.632
SVA	Follow-up	16.23±7.72	15.99±9.06	15.79±6.82	.277
	P	0.042	0.718	.015	
	P’	0.978	0.588	.999	
	Preoperative	20.11±6.08	20.08±5.83	20.13±6.25	.954
	Postoperative	17.48±5.96	18.36±6.13	16.97±5.82	.103
T1S	Follow-up	20.26±6.04	20.00±5.41	20.42±6.39	.623
	P	<0.001	.206	<0.001	
	P’	0.991	.999	.975	
	Preoperative	7.96±8.09	8.39±8.85	7.71±7.64	.556
	Postoperative	3.70±6.87	4.21±9.02	3.41±5.25	.418
TS-CL	Follow-up	5.14±7.67	4.67±7.03	5.41±8.03	.502
	P	0.001	.012	<0.001	
	P’	0.001	.013	.050	
	Preoperative	76.33±8.07	76.89±8.83	76.78±7.63	.923
	Postoperative	78.67±5.72	79.13±5.38	79.20±5.95	.924
SCA	Follow-up	75.75±6.74	74.98±7.02	77.04±6.53	.033
	P	0.004	.164	.012	
	P’	0.965	.358	.986	
	Preoperative	57.91±5.56	57.09±5.08	59.23±5.71	.006
	Postoperative	57.16±5.40	56.16±4.63	58.57±5.67	.002
NT	Follow-up	56.87±5.18	56.11±4.33	58.11±5.55	.007
	P	0.639	.546	.711	
	P’	0.252	.477	.280	
	Preoperative	78.59±7.25	78.00±7.59	79.73±7.03	.095
	Postoperative	77.50±6.52	75.78±5.39	79.26±6.80	<0.001
TIA	Follow-up	78.95±6.62	78.78±5.51	79.86±7.21	.257
	P	0.483	.106	.924	
	P’	0.996	.846	.998	
	Preoperative	20.84±6.17	20.92±5.87	20.79±6.35	.881
NDI score	Follow-up	16.52±6.90	16.29±6.36	16.66±7.20	.714
	p	<0.001	<0.001	<0.001	
BMI (Kg/m^2^)		23.78±2.62	23.15±2.43	24.15±2.67	.007
Sex (male/female)		147/65	62/16	85/49	.014
Age (years)		52.59±10.46	48.38±10.12	55.04±9.88	.000
Duration of symptoms (mouths)		9.38±16.95	9.04±18.28	9.58±16.19	.824

*Comparisons of preoperative, postoperative and follow-up cervical parameters, P was result of preoperative and postoperative comparison, P’ was result of preoperative and follow-up comparison*

In multi-segment group, postoperative SVA(*P* = 0.015) and T1S(*P* < 0.001) were smaller, and SCA(*P* < 0.001) was larger than preoperative. However, in single-segment group, there was no difference in postoperative T1S, SVA, and SCA compared with preoperative. Preoperative NT(*P* = 0.006), postoperative NT(*P* = 0.002), follow-up NT(*P* = 0.007), age(*P* < 0.001), sex(*P* = 0.014), and BMI(*P* = 0.007) of multi-segment group have differences with single-segment group.

### Correlation analysis of follow-up NDI scores, cervical sagittal parameters, and clinical data

There was a close correlation between sagittal parameters at the last follow-up, as shown in Table 4. T1S was positively correlated with CL (*r* = 0.245), SVA (*r* = 0.184), and negatively correlated with SCA (*r* = − 0.314) and NT (*r* = − 0.222). There was a significant correlation between SVA and TS-CL (*r* = 0.207), SVA and TIA (*r* = 0.322), and SVA and SCA (*r* = 0.167). In addition, SCA is closely related to CL (*r* = − 0.573), and TIA is highly correlated to T1S + NT (*r* = 0.425, *P* < 0.001).

**Table 4:** Correlation Between Radiographic Parameters and NDI Scores at the last follow-up

	CL	T1S	SVA	TS-CL	SCA	NT	TIA	NDI	Age	Sex	BMI	Time	Segment
CL	X	.245^**^	-.074	-.646^**^	-.573^**^	-.227^**^	.200^**^	.055	.064	-.014	-.027	.080	-.023
T1S		X	.184^**^	.582^**^	-.314^**^	-.222^**^	.098	.689^**^	.128	-.009	-.007	.004	.034
cSVA			X	.207^**^	.167^*^	-.053	.322^**^	.155^*^	.150^*^	.283^**^	.279^**^	-.132	-.075
TS-CL				X	.234^**^	.015	-.090	.496^**^	.047	.005	.018	-.063	.046
SCA					X	.349^**^	-.134	-.142^*^	.327^**^	.107	.200^**^	-.016	.147^*^
NT						X	.461^**^	-.072	.156^*^	-.073	.207^**^	.023	.174^*^
TIA							X	.047	.132	-.086	.291^**^	.037	.078
NDI								X	.194^**^	.095	-.004	-.029	.025
Age									X	-.120	.158^*^	.123	.308^**^
Sex										X	.124	-.078	-.168^*^
BMI											X	-.111	.184^**^
Time												X	.015
Segment													X

**Correlation is significant at the 0.01 level (2 tailed); *Correlation is significant at the 0.05 level (2 tailed)

*Time indicates duration of symptoms; Segment: single-segment indicates 0; multiple-segment indicates 1*

**Fig. 6:**
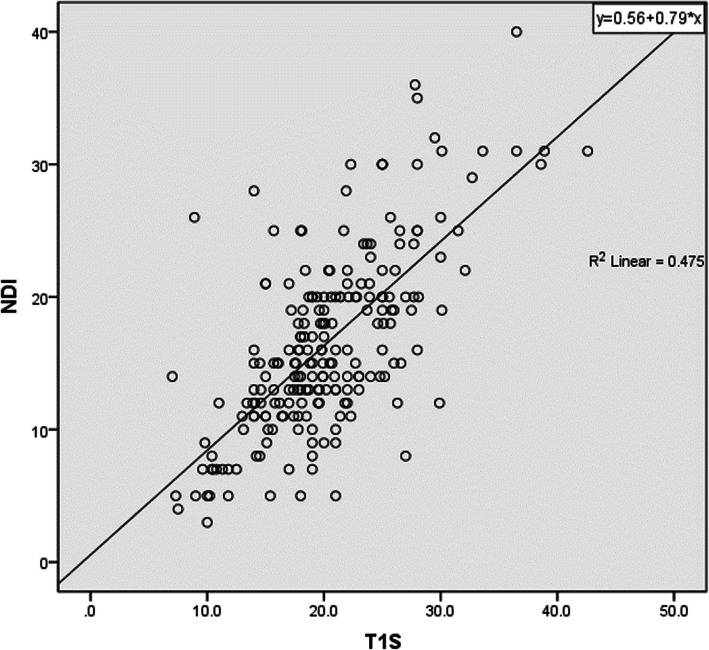
A positive correlation was observed between NDI scores and T1S values

**Fig. 7:**
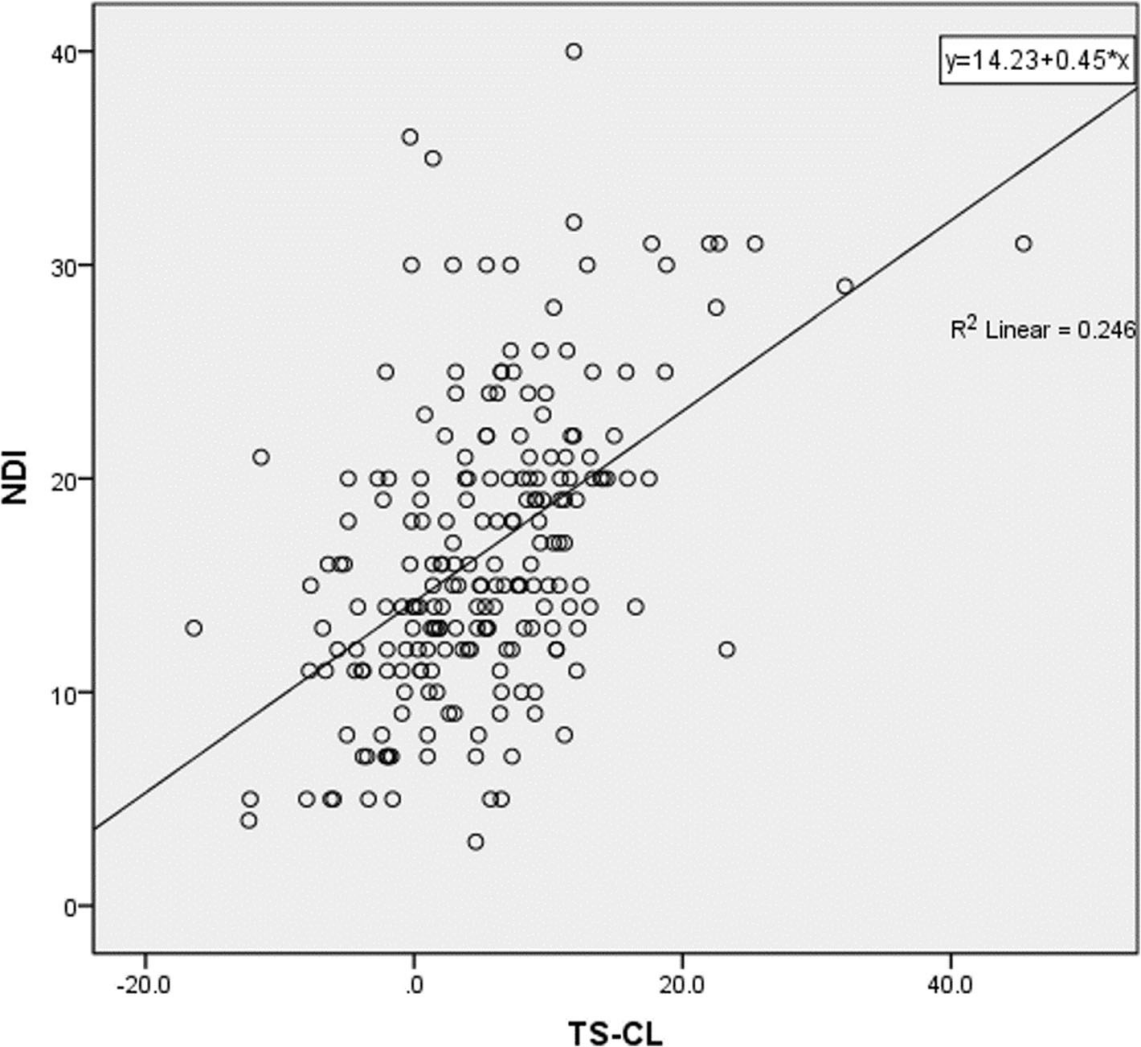
A positive correlation was observed between NDI scores and TS-CL values

The correlation analysis of follow-up NDI scores is shown in Table 4. NDI score was positively correlated with T1S (*r* = 0.689) (Fig. 6), SVA (*r* = 0.155), and TS-CL (*r* = 0.496) (as Fig. 7), while negatively correlated with SCA (*r* = − 0.142). There was no significant correlation between preoperative NDI score and CL, TIA, and NT. At the last follow-up, NDI score was positively correlated with age (*r* = 0.194), and no significant correlation with gender, BMI, single-segment or multi-segment fusion, or duration of symptoms. Operative segment (single-segment ACDF or multi-segment ACDF) was associated with SCA(*r* = 0.147), NT(*r* = 0.174), sex(*r* = − 0.168), age(*r* = 0.308), and BMI(*r* = 0.184).

### Multiple regression analysis of NDI score

According to the multiple regression and linear regression analysis of NDI score (Table 5), T1S (*B* = 0.809, *P* < 0.01), CL (*B* = − 0.152, *P* = 0.30), sex (*B* = 1.962, *P* = 0.016), and age (*B* = 0.110, *P* = 0.06) were linearly dependent on NDI scores. A linear regression model was established with the following formula: NDI =0 .809 × (T1S) − 0.152 × (CL) + 1.962 × (Sex) + 0.110 × (Age).

**Table 5:** Multiple regression analysis and linear regression of NDI score

	B	Standardized Coefficients	Sig
T1S	0.809	0.707	.000
SVA	-0.011	-0.012	.847
SCA	-0.084	-0.082	.288
CL	-0.152	-0.142	.030
NT	0.130	0.098	.159
TIA	-0.046	-0.044	.541
Age	0.110	0.166	.006
Sex	1.962	0.131	.016
Single or multiple segments	-0.402	-0.028	.598
Duration of symptoms	-0.015	-0.038	.455
Constant	0.511		.947

*Sex: male indicates 1; female indicates 0*

*Single-segment indicates 0; multiple-segment indicates 1*

As shown in Table 6, 82 patients in group 1 were less than 50 years old. NDI score was correlated with T1S(*r* = 0.658), TS-CL(*r* = 0.544), and SCA(*r* = − 0.303). In group 2, 82 patients were aged 50–60 years, and NDI score was correlated with T1S(*r* = 0.581) and TS-CL(*r* = 0.436). Group 3 patients were over 60 years old, with a total of 48 patients. NDI score was correlated with T1S(*r* = 0.717) and TS-CL(*r* = 0.363).

**Table 6:** Correlation analysis of follow-up cervical parameters and NDI score by age group

	Group1		Group2		Group3	
	r	p	r	p	r	p
C2-7 Cobb angle	0.053	0.635	0.166	0.137	0.128	0.385
T1S	0.658	<0.001	0.581	<0.001	0.717	<0.001
cSVA	0.015	0.890	0.187	0.093	0.120	0.416
TS-CL	0.544	<0.001	0.436	<0.001	0.363	0.011
SCA	-0.303	0.006	-0.211	0.057	-0.195	0.184
NT	-0.029	0.793	-0.022	0.843	0.016	0.912
TIA	0.152	0.173	0.031	0.781	-0.078	0.600

*Group1: age<49 years, Group2: age 50-60 years, Group3: age>60 years*

### Analysis of risk factors for last follow-up NDI score deterioration

Whether the follow-up NDI score was higher than preoperative NDI was used as the dependent variable for logistics regression. The final result is shown in Table 7. TS-CL was strongly correlated with T1S and CL, so it was not included in the analysis. T1S (*B* = 0.205, *P* < 0.001), CL (*B* = − 0.094, *P* = 0.041) and NT (*B* = 0.142, *P* = 0.023) were independent risk factors that affected whether the last follow-up NDI score was greater than preoperative.

**Table 7:** risk factors analysis of NDI score worsen at the last follow-up

	B	SE	Wald	P value	OR value	Confidence interval of 95% EXP(B)	
						minimum	maximum
T1S	0.205	0.046	20.124	<0.001	1.227	1.122	1.342
SVA	0.046	0.039	1.378	0.240	1.047	0.970	1.129
SCA	-0.026	0.051	0.263	0.608	0.974	0.882	1.077
CL	-0.094	0.046	4.169	0.041	0.910	0.831	0.996
NT	0.142	0.062	5.202	0.023	1.152	1.020	1.301
TIA	0.011	0.048	0.049	0.825	1.011	0.919	1.111
Age	-0.010	0.027	0.128	0.720	0.990	0.940	1.044
Sex	-0.011	0.526	0.000	0.983	0.989	0.353	2.774
Single or multiple segments	0.012	0.489	0.001	0.981	1.012	0.973	1.027
Duration of symptoms	0.000	0.014	0.001	0.974	1.000	0.831	0.996

*Sex: male indicates 1; female indicates 0*

*Single-segment indicates 0; multiple-segment indicates 1*

### Reliability analysis

The assessment of intra-observer and inter-observer reliability for cervical and thoracic parameters showed excellent consistency, respectively (Table 8).

**Table 8:** Inter-observer Reliability and Intra-observer Reproducibility Using the Intraclass Correlation Coefficient*

	Inter-observer	Intra-observer
T1S	0.87	0.81
CL	0.79	0.72
SVA	0.88	0.82
SCA	0.90	0.86
TS-CL	0.86	0.83
NT	0.85	0.76
TIA	0.82	0.79

*An intraclass correlation coefficient value of <0.6 indicates poor reliability; 0.6–0.79 indicates good reliability; and 0.8–1.0 indicates excellent reliability

## Discussion

This study found that CL, SVA, T1S, and TS-CL were significantly improved after ACDF, while NT and TIA were relatively stable and not affected by ACDF, which was basically consistent with previous studies [4–6]; SCA, as a new parameter, increases in the postoperative measurement, while follow-up SCA is basically the same as preoperative, with no significant change. In addition, we also found that at the last follow-up, some parameters which improved significantly after surgery gradually changed to preoperative state, or even worse than preoperative. SVA and T1S improved significantly after surgery, but the orthopedic effect was lost at the last follow-up, which was close to preoperative level. It may be related to the changes in bearing weight and angle of adjacent segments, especially the lower cervical, after ACDF [7]; after fusion, the lower cervical and the intervertebral disc bear more weight, and due to orthopedic changes in the distribution of bearing angle and force, there are compensatory changes in the lower cervical, resulting in an increase in T1S compared with the preoperative level. In addition, we found that T1S, SVA, and SCA improved obviously after surgery in multi-segment group, while single-segment group showed no obvious improvement. Multi-segment ACDF can better improve cervical parameters and restore cervical sagittal balance. However, after multi-segment ACDF, cervical stability is poorer and the adjacent stage disease (ASD) incidence is higher than single segment ACDF.

As for the evaluation of postoperative ACDF efficacy, commonly used methods include pain-related VAS score, cervical JOA score, and cervical NDI score. Studies have compared the efficacy of each scoring method in evaluating the efficacy of cervical orthopedic surgery [8]. Accordingly, cervical NDI score was used to evaluate postoperative spinal cord improvement and patients’ quality of life. Because of the long recovery period of CSM, we used the last follow-up NDI scores to compare with that before surgery. After ACDF, NDI score was significantly improved compared with those of preoperative, but some patient follow-up NDI scores increase compared to preoperative NDI. In our study, whether the NDI score increased after surgery was used as dependent variables, and cervical parameters was used as independent variables, performing a binary logistic regression analysis.

The thoracic 1 vertebra is the bridge between cervical and thoracic vertebra. T1S is a very important parameter of cervical segment, which is closely related to other parameters as a bond [9]. T1S is not only closely correlated with CL and SVA, but also with NT. In addition, it is negatively correlated with SCA. Changes in T1S may reflect changes in the sagittal balance of cervicothoracic spine. Studies found that [10] there is a strong correlation between T1S and other parameters, and T1S changes earlier when cervical balance is destroyed. The majority of cervical imbalance is caused by disc herniation, neck muscle fatigue, surrounding ligament relaxation, and the dislocation of vertebral body alignment caused by long-term bending. These reasons may also lead to the increase of SVA and the decrease of CL. Studies [11] have found a significant negative correlation between C2–7 Cobb angle and cervical SVA. In asymptomatic Chinese population, CL was significantly correlated with other cervical sagittal parameters including TIA, T1S, NT, and C2–7 SVA [12].

SCA, a new parameter introduced in recent years, is mainly used to reflect the balance of the head and neck spine. SCA has a high correlation with T1S and CL, and a weak correlation with SVA, which is the link between cervical segment and cranium. It was found in previous studies [13] that TIA was approximately equal to the sum of T1S plus NT, which was also confirmed in this study. TIA and NT were not sensitive to surgical changes, and the postoperative changes of T1S were relatively large. However, this equation was valid both before and after surgery. When T1S changes significantly, TIA and NT make corresponding adjustments to adapt. This allows the balance of the upper spine to be maintained with minimal changes.

Studies [2, 14] found a correlation between NDI score and cervical parameters. Preoperative NDI score increased with the increase of C2–7 SVA and TS-CL, while high C2–7 SVA and low TIA were independent predictors of high preoperative NDI score. Preoperative T1S was positively correlated with preoperative NDI score, and postoperative follow-up T1S was positively correlated with follow-up NDI score. In addition, C2–7 SVA, and TS-CL were positively correlated with NDI score respectively. It can be considered that cervical sagittal parameters are closely related to NDI score. Cervical parameters can be used to evaluate the prognosis of patients after ACDF. Oversize T1S, SVA, and mismatched TS-CL will have adverse effects on the prognosis. This study also found a negative correlation between NDI score and SCA. SCA is an important parameter for maintaining sagittal balance of cervical spine and maintaining a normal value of 83° ± 9° can promote good prognosis [15].

It is believed that cervical curvature has an impact on surgical prognosis, and the improvement of cervical lordosis will affect the long-term efficacy of ACDF in the treatment of degenerative cervical disc disease [16]. Kyphosis of the cervical can affect the outcome of surgery. Cervical kyphosis is associated with increased neck pain before and after cervical surgery [17]. In this study, decreased cervical lordosis was a risk factor for deterioration of the last follow-up NDI score. Existing researches suggest that [18] patients with kyphosis are 18 times more likely to have cervical symptoms, a risk factor for neck pain. In addition, kyphosis may become a risk factor for spinal cord compression after cervical orthopedic surgery. Maintaining or reconstructing cervical kyphosis after ACDF surgery is helpful to obtain a good prognosis, and correcting kyphosis deformity as far as possible is still an accepted surgical option.

This study found that excessive T1S and NT were risk factors for follow-up NDI score greater than preoperative, which may lead to poor prognosis. Studies [3] have found that there is a linear relationship between T1S and postoperative NDI score. When T1S > 40° will lead to poor prognosis, T1S can be used as a parameter to evaluate prognosis. In addition, increased SVA will also affect the surgical effect [19]. The increase of T1S is often accompanied by the deterioration of SVA. If they change together, a poor prognosis will be the result. In addition to the consideration of contact with spinal cord compression and decompression, the correction of cervical imbalance and the prevention of T1S gradual deterioration after surgery are also factors to be considered. T1S is highly correlated with other parameters and tends to deteriorate after surgery, which will lead to poor prognosis. Therefore, more attention should be paid to this parameter in the future.

Our study found that T1S is positively correlated with CL. Excessive T1S leads to the compensatory increase of CL, which can maintain the horizontal gaze and relieve symptoms. However, due to cervical imbalance, intervertebral disc degeneration, hyperosteogeny, and muscle weakness, some cases did not have compensatory changes. Their postoperative T1S increased, but CL did not increase or even decreased. The result was an increase in follow-up NDI score.

Advanced age is associated with increased NDI score, which may relate to a number of reasons. It was found that C0–7 cervical lordosis, C2–7 cervical lordosis, and T1S were associated with age change [20]. In the elderly patients, cervical degeneration is severe and re-imbalance is likely to occur after surgery, leading to poor prognosis. The recovery rate of spinal cord in elderly patients is slower than that in middle-aged patients, and the clinical symptoms caused by long-term compression are not easy to recover, which affects the postoperative quality of life of patients. In this study, the correlation analysis of cervical parameters and NDI score was conducted in group according to age. We found that, compared with other two groups, the correlation between T1S and NDI score was stronger in the eldest group, and the correlation coefficient between other parameters and NDI score was smaller. In elderly patients, increased T1S is more likely to lead to increased NDI. However, they lack the compensatory ability for increased T1S.

## Conclusion

ACDF treatment of CSM, there was closely correlation between cervical sagittal parameters and NDI scores. T1S, CL, sex, and age were linearly dependent on NDI scores. The increase of T1S, NT, and the decrease of CL were independent risk factors that affected follow-up NDI score greater than preoperative. Cervical sagittal parameters were important in assessing the prognosis of ACDF. Reducing T1S is beneficial to clinical recovery.

## Data Availability

The data and materials have been uploaded to database as Supplementary Material.

## Abbreviations

ACDF: Anterior cervical decompression and fusion
CSM: Cervical spondylotic myelopathy
CL: Cervical lordosis
T1S: Thoracic 1 slope
SVA: Sagittal vertical axial
TS-CL: Thoracic 1 slope minus cervical lordosis
SCA: Spine-cranial angle
TIA: Thoracic inlet angle
NT: Neck tilt
NDI score: Neck Disability Index score
BMI: Body mass index
HRQOL: Health-related quality of life
